# Rationality of Time-Dependent Antimicrobial Use in Intensive Care Units in China: A Nationwide Cross-Sectional Survey

**DOI:** 10.3389/fmed.2021.584813

**Published:** 2021-02-17

**Authors:** Jiao Liu, Sheng Zhang, Sisi Huang, Yizhu Chen, Lidi Zhang, Hangxiang Du, Tao Wang, Yongan Liu, Yan Xu, Dechang Chen

**Affiliations:** ^1^Department of Critical Care Medicine, Ruijin Hospital, Shanghai Jiao Tong University School of Medicine, Shanghai, China; ^2^Department of Critical Care Medicine, Ruijin Hospital North, Shanghai Jiao Tong University School of Medicine, Shanghai, China

**Keywords:** extended infusion, continuous infusion, therapeutic drug monitoring, antibiotic stewardship team, time-dependent antibiotics

## Abstract

**Background:** Extended/continuous infusion and therapeutic drug monitoring (TDM) of time-dependent antimicrobials are recommended for optimizing drug exposure for patients in intensive care units (ICUs), although practical application of these measures remains uncertain. We surveyed current practices in infusion and monitoring of commonly prescribed time-dependent antimicrobials in ICUs across China.

**Methods:** From December 2019 to January 2020, we sent online questionnaires about various aspects of infusion and monitoring of time-dependent antimicrobials to intensivists across China. Responses from clinicians were matched with their professional titles using the Sankey diagram. Univariate and multivariate logistic regression analyses were performed to find factors associated with TDM.

**Results:** A total of 3,687 ICU specialists from 31 provincial administrative regions of China responded to our questionnaires. Antibiotic stewardship (ABS) teams were available in hospitals as reported by 3,243 (88.0%) intensivists, including 1,308 (35.5%) who were ABS team members. Although most intensivists (3,490, 94.7%) were acquainted with the concept of prolonged/continuous infusion, nearly half of them (1,634, 44.3%) commonly administered β-lactam antibiotics intermittently. Nearly two-thirds of the respondents reported that their hospitals could not perform TDM. Our multivariable logistic regression analysis revealed that at the hospital level, knowledge of drug sample timing and attitude toward monitoring treatment effects, and drug trough or peak concentration influenced the decision to conduct TDM.

**Conclusions:** We found great variability in prescribing practices, from drug administration to TDM, for several time-dependent antibiotics commonly used for patients with severe infections. Further studies are necessary to effectively evaluate strategies to promote consistent prescribing behavior.

## Introduction

Frequent inappropriate use of antibiotics could cause a bacterial resistance crisis. A recent estimate predicted that antimicrobial resistance may lead to 10 million deaths per year by 2050 (1). Antimicrobial therapy plays a key role in the treatment of bacterial infections in critically ill patients. Structured teams for promoting the sustainable use of antibiotics to ensure effective therapy (named antibiotic stewardship [ABS] teams) are crucial for curbing bacterial resistance (2). Intensive care units (ICUs) constitute major risk areas for the emergence of antimicrobial resistance. Studies have shown that ABS teams in ICUs can reduce antibiotic consumption, minimize the development of resistance, and shorten ICU stays (3, 4). Optimization of antibiotic administration based on pharmacokinetic (PK) and pharmacodynamic (PD) properties and therapeutic drug monitoring (TDM) is the key role of ABS, especially for time-dependent antibiotics (5). Time-dependent antibiotics eradicate microbes based on the time for which bacteria are exposed to the antibiotics at a concentration higher than the minimum inhibitory concentration (MIC). Administering antibiotics through prolonged infusion (extended or continuous) is the best way to meet PK/PD objectives (6). Patients in ICUs are frequently subjected to substantial PK variations that expose them to underdosing or overdosing, leading to treatment failure (7, 8). In a multinational PK point-prevalence study of eight β-lactam antibiotics in 248 patients, 16% of the patients did not achieve even the most conservative target concentration exposure (9).

Nevertheless, the strategy of measuring the plasma concentration of time-dependent antibiotics and using antibiotics based on optimal PK/PD has not yet been widely realized in real-world hospital settings. A multinational clinical survey of 402 professionals from 53 countries showed a tremendous variability in antibiotic administration. For instance piperacillin/tazobactam, vancomycin, and meropenem were administered by intermittent infusion in 72.4, 68.7, and 67% of ICU patients, respectively, while serum concentration was monitored in 1, 74, and 2% of ICU patients, respectively (10).

Hence, we aimed to survey a large sample of intensivists in China to understand current practices in administration and monitoring of commonly prescribed time-dependent antimicrobials including cefoperazone/sulbactam, piperacillin/tazobactam, carbapenems (meropenem and imipenem/cilastatin sodium), and vancomycin.

## Methods

### Study Protocol

We conducted a nationwide cross-sectional survey using an online questionnaire to assess the administration and TDM of time-dependent antibiotics according to their PK/PD properties in China. This was a negligible-risk study and, therefore, no ethical approval was required. The questionnaire was developed by intensivists based on recent recommendations and guidelines (11, 12) and tested on physicians in ICUs before it was validated. From December 17, 2019, to January 17, 2020, we sent open invitations to intensivists from all over the country to answer the survey questions. Weekly reminders were sent as required. The data from the anonymous responses were collected, uploaded into a Microsoft Excel file, and independently reviewed by two investigators.

### Questionnaire

The questionnaire comprised 38 questions with open and closed answers with multiple-choice options. The first part included 11 questions regarding the demographic and professional details of the respondents. Next, the respondents answered questions about their practice of and perceptions about time-dependent antibiotics infusions. The strategies for administering the antibiotics infusion were categorized as intermittent (≤ 30 min), extended (2–4 h), and continuous (24 h).

Six standardized clinical vignettes were used to assess how the respondents practiced these strategies. Four commonly used antimicrobials or antimicrobial classes were selected: cefoperazone/sulbactam, piperacillin/tazobactam, carbapenems (meropenem and imipenem/cilastatin sodium), and vancomycin. The participants responded to the questions based on the description of a sepsis patient weighing 60 kg with normal renal function. In the final section, we investigated the intensivists' knowledge of antibiotic serum concentration and their attitude toward TDM.

### Statistical Analyses

Sankey diagrams, which enable intuitive visualization, were used to match the responses with the professional titles of the respondents. Sankey diagrams start from the specification of the possible input files, then integrate the information into a web page, and finally generate the web page (13). Diagrams generated on specific web pages can be viewed on both Linux and Windows. The respondents were categorized as “TDM” or “no TDM” according to their responses to the questionnaire. Univariate and multivariable logistic regression analyses were performed to find factors associated with conducting TDM using the respondents' characteristics and their knowledge of TDM as covariates.

To avoid omitting useful variables, all covariates were included in the multivariable regression. We checked for adequate overlap in these factors between groups using a cross-validation model. To achieve this (14), the respondents in the database were divided into two subsets: a “training set” with 2,581 respondents (70%) and a “validation set” with 1,106 respondents (30%). The data from the survey were subject to descriptive statistical analyses using R software (version 3.6.2). Categorical variables were expressed as quantities and percentages. All statistical tests were two-sided, and differences with *P* < 0.05 were considered statistically significant.

## Results

### Respondent Characteristics

**Figure 1:**
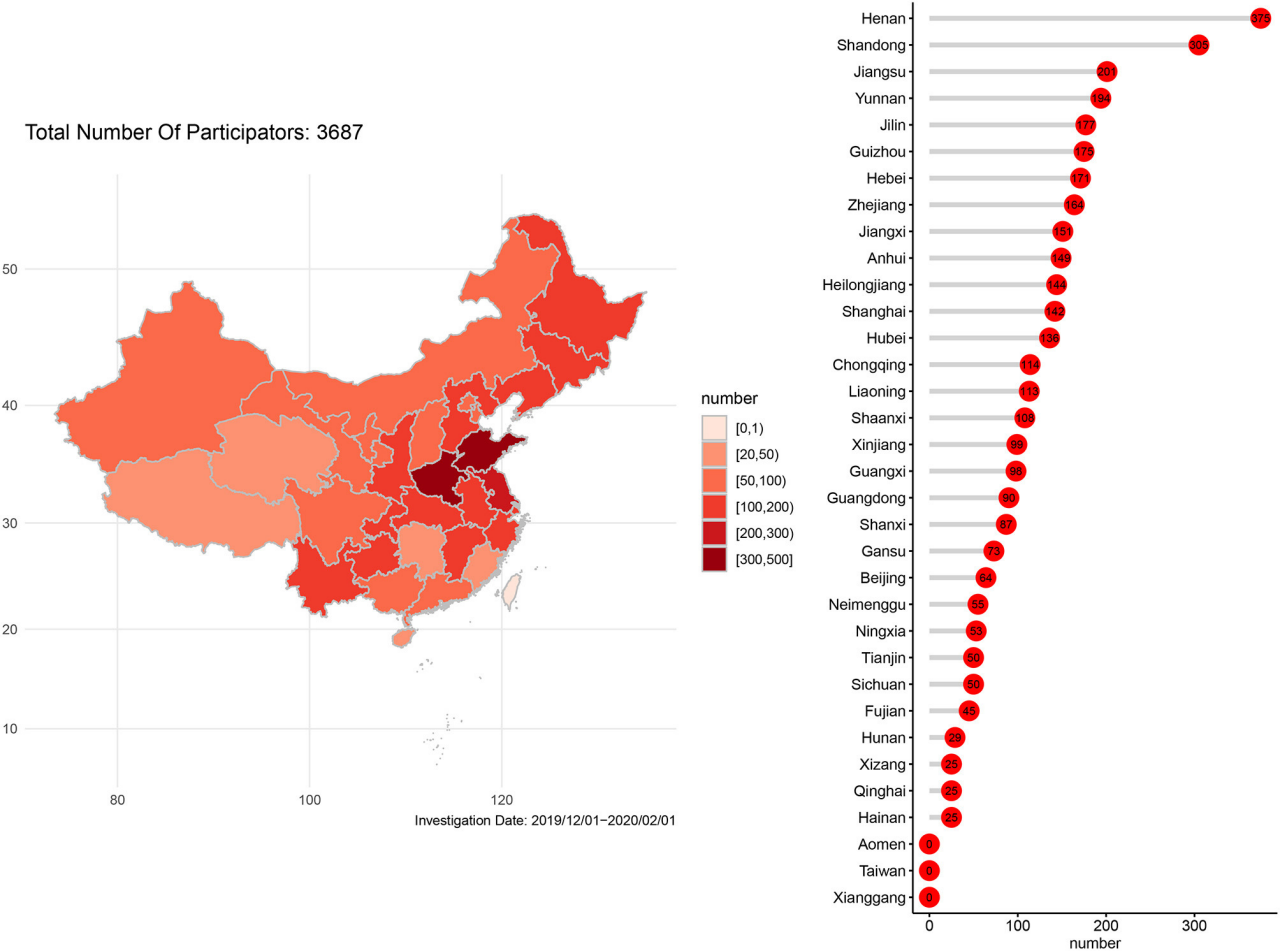
Geographical distribution of participants in China.

A total of 3,687 specialists in intensive care medicine from 31 provincial administrative regions of China responded to the questionnaire. The number of questionnaires that were sent out was 3,789, and the number of reply was 3,687; the reply rate was 97.3%. Personnel distribution among the respondents is shown in Figure 1. Demographic characteristics of the respondents are reported in Table 1.

**Table 1:** Demographics of survey participants.

**Variables**	**Percentage (%)**	***n/N***
**ICU type**		
ICU	85.54	3,154/3,687
EICU	5.75	212/3,687
Specialist ICU	8.71	321/3,687
**Age (years)**		
<30	12.1	449/3,687
30–40	47.1	1,735/3,687
40–50	32.2	1,186/3,687
50–60	8.5	313/3,687
>60	0.1	4/3,687
**Sex**		
Men	60	2,212/3,687
Women	40	1,475/3,687
**Degrees**		
Bachelor	49.8	1,835/3,687
Master	41.6	1,532/3,687
Doctor	8.1	300/3,687
others	0.5	20/3,687
**Hospital level**		
Tertiary	78.74	2,903/3,687
Secondary	21.23	783/3,687
Primary	0.03	1/3,687
**Position**		
Resident	20.4	751/3,687
Attending	35.4	1,306/,3687
Deputy chief	28.7	1,060/3,687
Chief	15.5	570/3,687
**On duty**		
Front-line	56.6	2,088/3,687
Second-line	28.6	1,053/3,687
Third-line	14.8	546/3,687

*ICU, intensive care unit; EICU, emergency intensive care unit; n, numbers; N, total numbers*.

### ABS Teams

ABS teams were available in hospitals reported by 3,243 (88.0%) respondents, including 1,308 (35.5%) who were members of ABS teams. We also explored the constitution of the ABS teams. Our results showed that 2,513 (68.2%) intensivists confirmed that clinical pharmacists directed the prescription of antibiotics and 1,825 (49.5%) intensivists confirmed that microbiologists interpreted the results of drug sensitivity tests (Table 2).

**Table 2:** Awareness of participants about antibiotic administration.

	**Percentage (%)**	***n/N***
**Knowledge about ABS teams**		
Is there a microbiologist to interpret the drug sensitivity results?		
Yes	49.5	1,825/3,687
Is there a clinical pharmacist to interpret the drug sensitivity results?		
Yes	68.2	2,513/3,687
Does your hospital have an ABS team?		
Yes	88	3,243/3,687
Are you a member of the ABS team?		
Yes	35.5	1,308/3,687
**Knowledge about time-dependent antibiotic infusion methods**		
Do you understand the concept of extended or continuous infusion of time-dependent antibiotics?		
Yes	94.7	3,490/3,687
When using time-dependent antibiotics such as β-lactam antibiotics, which is the most commonly used infusion method?		
Intermittent infusion	44.3	1,634/3,687
Extended infusion	39.3	1,448/3,687
Continuous infusion	4.1	152/3,687
Depending on the illness	12.3	453/3,687
Extended/continuous infusion is more effective than intermittent infusion when treating severe infections or sepsis with time-dependent antibiotics		
Agree	87	3,209/3,687
Disagree	1.8	66/3,687
Not sure	11.2	412/3,687
A prospective study on the 28-day mortality of patients with severe infection/sepsis treated with extended/continuous infusion and those treated with intermittent infusion would be valuable		
Agree	83.5	3,079/3,687
Disagree	1.49	55/3,687
Not sure	15	553/3,687
Knowledge about antibiotic TDM^*^		
If your hospital can measure drug concentration, will you do?		
Do for all patients	17.0	625/3,687
Depending on the illness	83.0	3,062/3,687
Do you think the interpretation of TDM results by clinical pharmacists is more rational for individualized antibiotic use?		
Yes	89.0	3,283/3,687

* *More responses about antibiotic TDM are presented in Table 3. ABS, antibiotic stewardship; TDM, therapeutic drug monitoring; n, numbers; N, Total numbers*.

### Method of Administration of Time-Dependent Antibiotics

**Figure 2:**
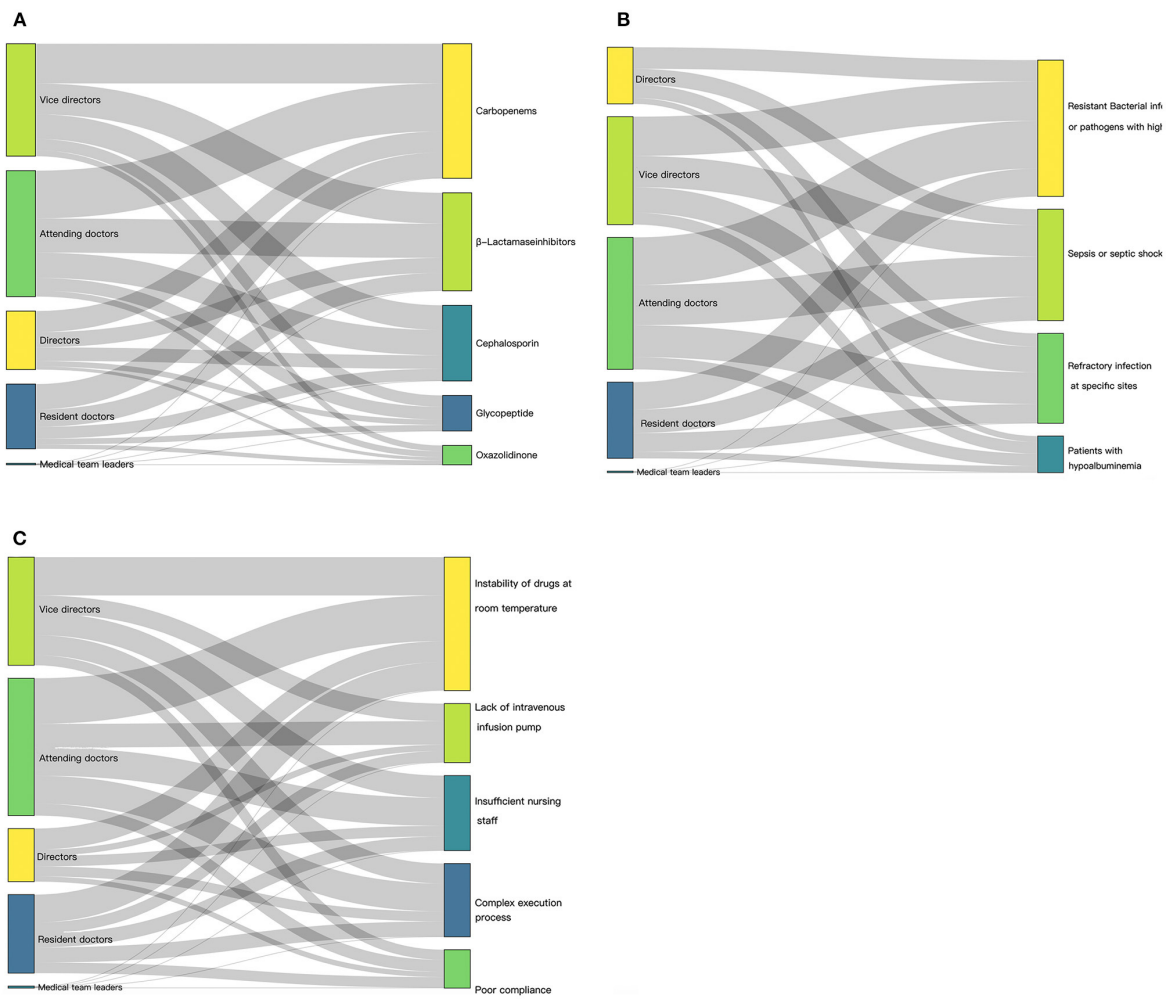
Illustration of current extended/continuous infusion practices, visualized as Sankey diagram. **(A)** Responses to whether different antibiotics are suitable for prolonged infusion matched with professional titles of respondents. **(B)** Main factors that influenced decision to change antibiotic infusion from intermittent to prolonged/continuous matched with professional titles of respondents. **(C)** Reason given for not choosing prolonged/continuous infusion matched with professional titles of the respondents.

Most intensivists (3,490, 94.7%) were acquainted with the concept of prolonged or continuous infusion of time-dependent antibiotics. However, nearly half of them (1,634, 44.3%) commonly administered time-dependent antibiotics such as β-lactam antibiotics intermittently. The majority (3,209, 87.0%) of participants agreed that prolonged or continuous infusion was more effective than intermittent infusion for the treatment of severe infection or sepsis. In addition, 3,079 (83.5%) intensivists agreed that a prospective study on 28-day mortality of patients with severe infection/sepsis who were treated with prolonged or continuous infusion would be valuable (Table 2). Our overall visual design followed the principle of the Sankey diagram, which links responses with professional titles by lines and signifies the quantities by line width (Figure 2). Most intensivists (3,370, 94.1%) chose carbapenems when asked which antibiotics can be administered using the prolonged infusion method. This included 995 vice directors, 529 directors, 1,198 attending doctors, 636 residents, and 12 medical team leaders.

Further, more than half of the respondents opined that β-lactamase inhibitors (2,454, 66.6%) and cephalosporin (1,894, 51.4%) can be administered through prolonged infusion. However, only 888/3,687 and 484/3,687 respondents deemed that glycopeptides and oxazolidinone, respectively, could be administered through prolonged infusion (Figure 2A). Further details for the different professional titles can be obtained by clicking on the corresponding areas of Figure 2. Prolonged infusion of antibiotics depended on the different hospital grade (Supplementary Figure 1). Most intensivists from secondary and tertiary hospitals chose carbapenems, β-lactamase inhibitors, and cephalosporin.

The main factors that influenced changing an antibiotic infusion from intermittent to prolonged/continuous were resistant bacterial infections or pathogens with high MIC (3,125, 84.8%), sepsis or septic shock (2,551, 69.2%), and refractory infections at specific sites (2,060, 55.9%), and a minority of respondents choosing hypoalbuminemia (837, 22.7%) (Figure 2B). When asked for reasons for not choosing prolonged/continuous infusion, most respondents mentioned medical conditions rather than patient compliance; the instability of drugs at room temperature was a particularly common cause (2,464, 66.8%) (Figure 2C).

### Clinical Vignettes

Most respondents (67.72%) reported administering extended courses of cefoperazone sulbactam to patients with hospital-acquired pneumonia (HAP)/ventilator-associated pneumonia (VAP) caused by multidrug resistant (MDR) *Acinetobacter baumannii*. Further, 63.55% of the respondents administered extended courses of piperacillin tazobactam to patients with HAP/VAP caused by MDR *Pseudomonas aeruginosa*. For meropenem or imipenem–cilastatin sodium in the case of complex abdominal infections, most of the respondents chose extended infusion, irrespective of the pathogenicity.

**Figure 3:**
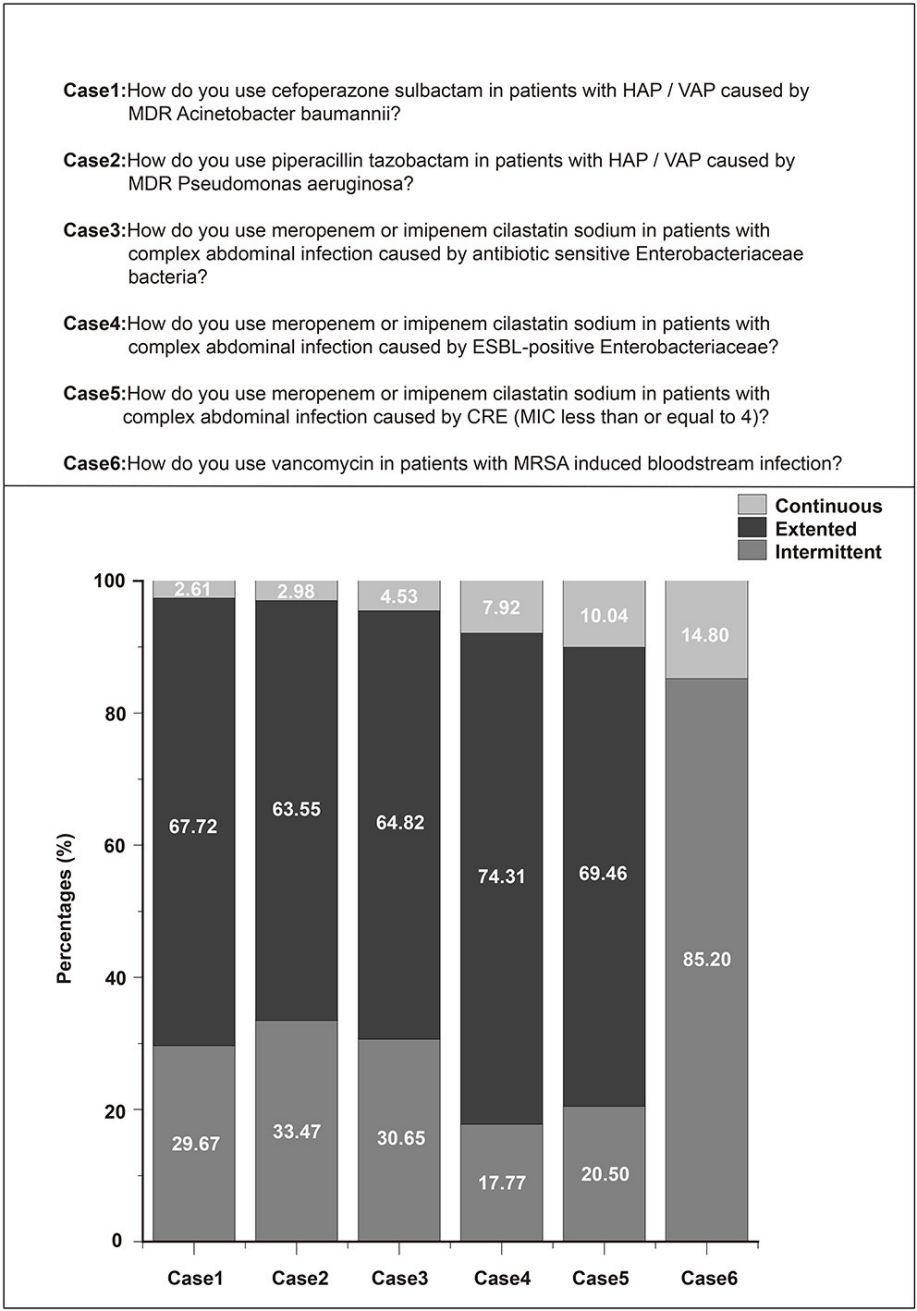
Current clinical practices in antibiotic administration.

Enterobacteriaceae included antibiotic-sensitive, extended-spectrum beta-lactamase (ESBL)-positive, or even carbapenem-resistant Enterobacteriaceae (CRE) (Figure 3). For the administration of vancomycin against methicillin-resistant *Staphylococcus aureus* (MRSA)-induced bloodstream infections, 85.2% of the respondents chose extended infusion and only 14.8% chose continuous infusion (Figure 3).

### TDM of Time-Dependent Antibiotics

**Figure 4:**
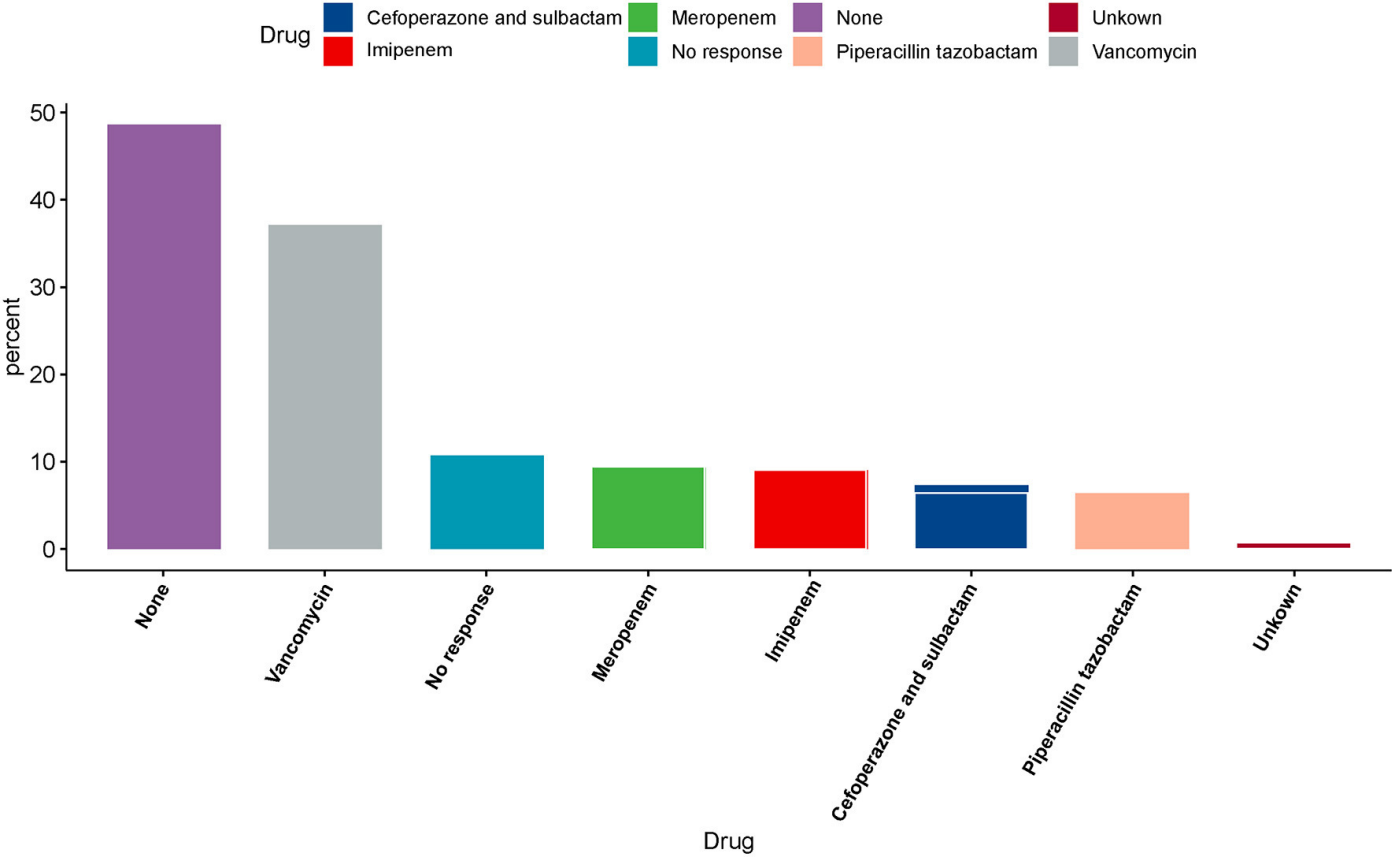
Illustration of antibiotics therapeutic drug monitoring (TDM) practices.

Nearly two-thirds of the respondents (2,317, 62.8%) reported that their hospitals could not perform TDM. Furthermore, we found that even if the hospital could implement TDM, only 17.3% intensivists would measure the drug concentration of every patient. Figure 4 shows responses about antibiotics that can be subjected to drug concentration measurement in the hospitals. Nearly half of the intensivists (1,792, 48.6%) confirmed that their hospital was incapable of measuring drug concentration. Except for 395 no responses, less than half (1,600, 43.4%) of the respondents reported that the hospital was capable of measuring drug concentration. In addition, vancomycin concentrations were predominantly measured in hospitals that could measure drug concentration (1,368, 37.1%) (Figure 4).

We found that nearly half of the respondents did not know the sample timings for trough and peak concentrations (1,541, 42%) or the valid ranges of trough (1,642, 45%) and peak concentrations (1869, 51%) (Table 3). Factors that influenced the intensivists' decision for conducting TDM were further investigated. Table 3 shows the results of our univariate logistic regression analysis. With the exception of department, professional title, and duty, all other variables were significantly different in the TDM and no TDM groups.

**Table 3:** Univariate analysis of factors associated with decision-making of intensivists on whether to perform therapeutic drug monitoring (TDM).

**Variables**	**Total (*n* = 3,687)**	**No TDM (*n* = 3,049)**	**TDM (*n* = 638)**	***p***
Department, *n* (%)				0.748
ICU	3,154 (86)	2,607 (86)	547 (86)	
EICU	212 (6)	179 (6)	33 (5)	
Specialist ICU	321 (9)	263 (9)	58 (9)	
Age, *n* (%)				0.05
<30	449 (12)	353 (12)	96 (15)	
30–40	1,735 (47)	1,425 (47)	310 (49)	
40–50	1,186 (32)	1,003 (33)	183 (29)	
50–60	313 (8)	264 (9)	49 (8)	
>60	4 (0)	4 (0)	0 (0)	
Sex, *n* (%)				<0.001
Male	2,212 (60)	1,871 (61)	341 (53)	
Female	1,475 (40)	1,178 (39)	297 (47)	
Degree, *n* (%)				<0.001
Others	20 (1)	17 (1)	3 (0)	
Bachelor	1,835 (50)	1,582 (52)	253 (40)	
Master	1,532 (42)	1,216 (40)	316 (50)	
Doctor	300 (8)	234 (8)	66 (10)	
Hospital level, *n* (%)				<0.001
Primary	1 (0)	1 (0)	0 (0)	
Secondary	783 (21)	691 (23)	92 (14)	
Tertiary	2,903 (79)	2,357 (77)	546 (86)	
Professional title, *n* (%)				0.059
Resident	751 (20)	609 (20)	142 (22)	
Attending	1,306 (35)	1,061 (35)	245 (38)	
Deputy chief	1,060 (29)	898 (29)	162 (25)	
Chief	570 (15)	481 (16)	89 (14)	
Duty, *n* (%)				0.059
Front-line	2,088 (57)	1,702 (56)	386 (61)	
Second-line	1,053 (29)	894 (29)	159 (25)	
Third-line	546 (15)	453 (15)	93 (15)	
Reasons for not conducting TDM, *n* (%)				<0.001
Conduct TDM	261 (7)	92 (3)	169 (26)	
Not available	2,317 (63)	1,920 (63)	397 (62)	
No severe infection or sepsis	365 (10)	340 (11)	25 (4)	
Depending on illness	333 (9)	325 (11)	8 (1)	
Infection by nonresistant bacteria	201 (5)	180 (6)	21 (3)	
No organ dysfunction	210 (6)	192 (6)	18 (3)	
Sample timing, *n* (%)				<0.001
Do not know	1,541 (42)	1,366 (45)	175 (27)	
Know	2,146 (58)	1,683 (55)	463 (73)	
Trough concentration and curative effect, *n* (%)				<0.001
Relevant	3,037 (82)	24,66 (81)	571 (89)	
Not relevant	142 (4)	116 (4)	26 (4)	
Do not know	508 (14)	467 (15)	41 (6)	
Peak concentration and curative effect, *n* (%)				0.001
Relevant	3,055 (83)	2,550 (84)	505 (79)	
Not relevant	178 (5)	130 (4)	48 (8)	
Do not know	454 (12)	369 (12)	85 (13)	
Trough concentration range, *n* (%)				<0.001
Do not know	1,642 (45)	1,430 (47)	212 (33)	
Know	2,045 (55)	1,619 (53)	426 (67)	
Peak concentration range, *n* (%)				<0.001
Do not know	1,869 (51)	1,599 (52)	270 (42)	
Know	1,818 (49)	1,450 (48)	368 (58)	
Pharmacist for TDM, *n* (%)				<0.001
No	2,068 (56)	1,773 (58)	295 (46)	
Yes	1,619 (44)	1,276 (42)	343 (54)	

*ICU, intensive care unit; EICU, emergency intensive care unit; TDM, therapeutic drug monitoring*.

Our multivariable logistic regression analysis revealed that intensivists who work in tertiary-level hospitals (odds ratio [OR]: 1.47, 95% confidence interval [CI]: 1.07–2.06) and know drug sample timing (OR: 1.80, 95% CI: 1.40–2.31) were more likely to choose to conduct TDM. Intensivists who lack the knowledge of drug trough concentration and curative effect (OR: 0.34, 95% CI: 0.20–0.55) and drug peak concentration and curative effect (OR: 1.86, 95% CI: 1.20–2.83) also limited their application of TDM. In addition, the reasons that intensivists chose for not conducting TDM also affected their application of TDM (Table 4).

**Table 4:** Multivariable analysis of factors associated with decision-making of intensivists on whether to perform therapeutic drug monitoring (TDM).

**Variables**	**OR**	**95% CI**	***p***
**Sex**			
Male (reference)	–	–	–
Female	1.22	0.97–1.53	0.129
**Hospital level**			
Secondary (reference)	–	–	–
Tertiary	1.47	1.07–2.06	0.046
**Duty**			
Front-line (reference)	–	–	–
Second-line	0.56	0.42–0.74	0.004
Third-line	0.89	0.65–1.20	0.661
**Reasons for not conducting TDM**			
Conduct TDM (reference)	–	–	–
Not available	0.15	0.10–0.22	<0.001
No severe infection or sepsis	0.02	0.01–0.05	<0.001
Depending on illness	0.03	0.01–0.07	<0.001
Infection by non-resistant bacteria	0.08	0.04–0.14	<0.001
No organ dysfunction	0.07	0.04–0.14	<0.001
**Sample timing**			
Do not know (reference)	–	–	–
Know	1.80	1.40–2.31	<0.001
**Trough concentration and curative effect**			
Relevant (reference)	–	–	–
Not relevant	1.56	0.90–2.63	0.094
Do not know	0.34	0.20–0.55	<0.001
**Peak concentration and curative effect**			0.001
Relevant (reference)	–	–	–
Not relevant	1.86	1.20–2.83	0.007
Do not know	1.27	0.88–1.82	0.189
**Pharmacist for TDM**			
No (reference)	–	–	–
Yes	1.25	0.97–1.60	0.173

*OR, odds ratio; 95% CI, 95% confidence interval; TDM, therapeutic drug monitoring*.

The presence of pharmacists (OR: 1.25, 95% CI: 0.97–1.60) did not seem to influence the decision of clinicians for performing TDM (Table 4). However, more than four-fifth of the respondents (3,283, 89.0%) agreed that the interpretation of TDM results by clinical pharmacists is better and more rational for individualized antibiotic use (Table 2).

## Discussion

To the best of our knowledge, this is the first nationwide study evaluating the rationality in the use of intravenous time-dependent antibiotics in ICUs in China. The majority of respondents confirmed the existence of ABS teams to improve the quality of antibiotic use, although interactions between ABS team members and intensivists may not be sufficient. In this survey, we found wide variability in prescribing practices, from drug administration to the use of TDM, for several time-dependent antibiotics commonly administered to patients with severe infections. We aimed to provide a basis for standardizing antibiotic use in clinical practice to ensure that minimal quality standards are met and that all patients benefit from it.

ABS should be performed as a multifaceted strategy to improve the quality of antibiotic use. Taggart et al. (15) showed that ABS programs in a trauma and neurosurgery ICU reduced the mean total monthly antimicrobial use by 28%. ABS teams guarantee the implementation of such ABS programs. Our survey revealed that most hospitals in China have ABS teams. Because of the complicated nature of antibiotic application, interdisciplinary teams comprising infection disease specialists, intensivists, clinical pharmacists, and microbiologists are necessary. However, only half the intensivists in our survey reported pharmacists and microbiologists in antibiotic prescription management. Thus, it is likely that ABS teams function suboptimally because of insufficient interactions between ABS team members and intensivists. It is also concerning that many hospitals had very few pharmacists or microbiologists. Thus, it is a pressing need to train more clinical pharmacists and clinical microbiologists and to include them in ABS teams.

Most of the intensivists in China surveyed in this study were acquainted with the concept of prolonged/continuous infusion, with 87.0% of them believing that it was more effective for treating severe infections or sepsis. However, less than half of the intensivists (43.4%) opted to administer β-lactam antibiotics in a prolonged/continuous manner, whereas nearly a third thought that β-lactamase inhibitors cannot be administered using prolonged infusion. These results may differ from those obtained in other geographic regions because of inherent differences in medical practice in different countries. For example, an observational study reported that more than half of the participants in Belgium and France received an extended or continuous infusion of β-lactam antibiotics (16). In the case of carbapenems such as meropenem or imipenem/cilastatin sodium, nearly two-thirds of our respondents prescribed them as extended or continuous infusions (Figure 3), which is a higher fraction than that reported in the ADMIN-ICU survey for 32% (10) and a survey from Australia and New Zealand for 26.1% (17). Only 14.8% of intensivists chose continuous infusion for vancomycin in patients with MRSA-induced bloodstream infections in this study. This may be due to nephrotoxicity caused by vancomycin. However, continuous vancomycin infusion has been associated with a low nephrotoxicity risk (18–20), but no clinical superiority compared with intermittent dosing. In contrast, in a nationwide cross-sectional survey in France (21), more than 95% of infectious disease specialists and intensivists chose continuous infusion of vancomycin, despite little evidence of superior efficacy and tolerance. Whether prolonged/continuous infusion is better than intermittent infusion still lacks evidence. Dulhunty et al. (22) conducted a multicenter randomized trial to evaluate the efficacy of intermittent vs. continuous infusion of β-lactam antibiotics in patients with severe sepsis and found no difference between two groups in clinical cure rates (49.5 vs. 52.4%).

One of the main reasons cited for not choosing extended/continuous infusion was the instability of drugs at room temperature, suggesting that it would be critical to improve pharmacological knowledge among intensivists. Many intensivists changed intermittent infusions to prolonged/continuous infusions for patients infected with pathogens with a high MIC or who developed sepsis, revealing a strong PK/PD rationale. However, hypoalbuminemia is often overlooked, with only 22.7% of intensivists in our survey opting to change the infusion method for patients with hypoalbuminemia (Figure 2B). Antibiotics with a high protein-binding rate may have significant effects in patients with hypoproteinemia, which occurs in 40% of critically ill patients (23).

TDM is commonly used to optimize the dose of a drug to meet the PK/PD objective for individual patients. Less than half of the respondents (43.4%) reported that their hospitals could monitor the concentration of antibiotics, mostly (37.1%) for vancomycin. This result differs from that of the ADMIN-ICU survey, which revealed that TDM is routinely applied, but mostly for aminoglycosides and glycopeptides (10). To improve efficacy and minimize toxicity for antibiotics, TDM measurement is necessary. Vancomycin is a time-dependent antibiotic with long post-antibiotic effects. To achieve the dosing scheme target, the ratio of area under the concentration–time curve within the 24-h period to MIC (AUC_0−24_/MIC) was over 400 (24). The expert guideline committee also recommended to maintain vancomycin trough concentrations between 15 and 20 mg/L as a surrogate marker of AUC_0−24_/MIC ≥400 for serious infections due to MRSA (25). Safely attaining an optimal AUC_0−24_/MIC ratio when treating pathogens with MICs of >1 mg/L is highly challenging (26). Dose delivery optimization requires measurement, and the trough concentration of vancomycin is widely measured clinically (24).

TDM for beta-lactams is not routinely used in regular practice. An initial loading dose followed by prolonged or continuous beta-lactam infusion is likely enough to achieve a PK/PD target and improve clinical outcomes in critically ill patients (27, 28). Myelosuppression is a well-known side effect of beta-lactams (29); however, no toxic thresholds have been well-established to date and the peak concentration was not usually detected in the clinical works. TDM for beta-lactams in critically ill patients may improve poor prognosis and potentially reduce the emergence of resistance. Dose should be adjusted if the serum trough or steady-state level was below 5–5-fold the minimum inhibitory concentration (MIC) or above 10-fold MIC. The ADMIN-ICU survey found that only 1% of clinicians detected TDM for piperacillin/tazobactam (10). Only 21% of the doctors in the ANTIBIOPERF study survey measured the beta-lactam TDM (21). Less than 10% of intensivists in our study assumed the ability of beta-lactams TDM, such as meropenem, imipenem cilastatin sodium, cefoperazone sulbactam, and piperacillin tazobactam in their hospital (Figure 4). A prospective study explored the practicality of beta-lactam TDM measurement in critically ill patients. A total of 236 patients were subject to TDM measurement. Among them, 175 (74.2%) patients required dose adjustment. Unfortunately, few studies explored the outcome between patients with TDM or without TDM for beta-lactams. Further study is required to determine whether achievement of optimal beta-lactam pharmacodynamic targets could improve clinical outcomes or not (27).

We also noticed that nearly half the respondents in this survey did not know sample timings or valid ranges of trough and peak concentrations. Further, most respondents were also unaware of the concept of measuring “trough” and “peak” therapeutic drug concentrations to adjust dosing in real-world hospital settings. This phenomenon is not unique to China. In a prospective trial conducted in the US among 233 hospitalized adults whose plasma vancomycin concentrations were to be measured as trough concentrations, only 84 (36%) were actually sampled correctly (30). Comparing the concentration obtained outside the window with a pre-specified target range may lead to erroneous conclusions about drug dosage (31).

Our multivariable logistic regression analysis revealed that the decision to perform TDM is influenced by the hospital level, the knowledge of drug sample timing, and the attitude toward monitoring the drug trough or peak concentration and their clinical effects. We found that most respondents (774/783) in secondary hospitals could not measure drug concentration, which may be the most important factor. Apart from the lack of facilities in such hospitals, PK/PD data is also essential for TDM.

## Limitations

This study has a few limitations that need to be addressed in future studies. First, simplified and standardized clinical vignettes (one weight and normal renal function) were used to evaluate clinical practices homogeneously in our survey. Although clinical vignettes are deemed valid tools that are widely used to measure the quality of clinical practices (32), they may not accurately represent the complexity of the decision-making when a therapeutic strategy is being developed for a similar patient. Second, the questionnaire design may have been susceptible to acquiring duplicate or irrelevant data. For example, one of the questions enquired about the time required for obtaining drug concentration results after sending blood samples. As only one-third of the respondents reported that the hospitals were capable of measuring drug concentration, most of the data from this question was meaningless. Furthermore, for the manual way of collecting data, there was inevitable error during data collection. At last, the number of respondents from different grade of hospitals widely varied. It may cause inevitable bias.

## Conclusions

Despite the limitations discussed in the previous section, we believe that this survey critically highlights the significant variability in antimicrobial infusion and monitoring practices in ICUs in China. Our results indicate that one strategy to improve these practices is the improvement of ABS teams and strengthening of collaboration between specialists such as intensivists, microbiologists, and pharmacists. Another strategy would be to make prolonged infusion of time-dependent antimicrobials and TDM prime objectives of ABS programs to meet PK/PD objectives. Importantly, awareness about PK/PD should be urgently raised among intensivists. Future studies should aim to develop better guidelines, establish consistent prescribing behavior of time-dependent antibiotics, and encourage periodic evaluate the effectiveness of these strategies.

## Summary

We surveyed intensivists across China and found great variability in the prescription and monitoring of popular time-dependent antibiotics. Consistent prescribing practices, improved pharmacological awareness, and interdisciplinary efforts are necessary for the sustainable use of antibiotics in the future.

## Data Availability Statement

The original contributions presented in the study are included in the article/Supplementary Material, further inquiries can be directed to the corresponding author/s.

## Ethics Statement

Ethical review and approval was not required for the study on human participants in accordance with the local legislation and institutional requirements. Written informed consent for participation was not required for this study in accordance with the national legislation and the institutional requirements.

## Author Contributions

All authors listed have made a substantial, direct and intellectual contribution to the work, and approved it for publication.

## Conflict of Interest

The authors declare that the research was conducted in the absence of any commercial or financial relationships that could be construed as a potential conflict of interest.

## Supplementary Material

Supplementary Figure 1 Prescribing preferences of antibiotics among hospitals at different grades.
