# Supplementary figures and images for: Rationality of Time-Dependent Antimicrobial Use in Intensive Care Units in China: A Nationwide Cross-Sectional Survey
Source: Front Med (Lausanne). 2021 Feb 17;8:584813. doi: 10.3389/fmed.2021.584813 (PMC7925833; doi:10.3389/fmed.2021.584813)

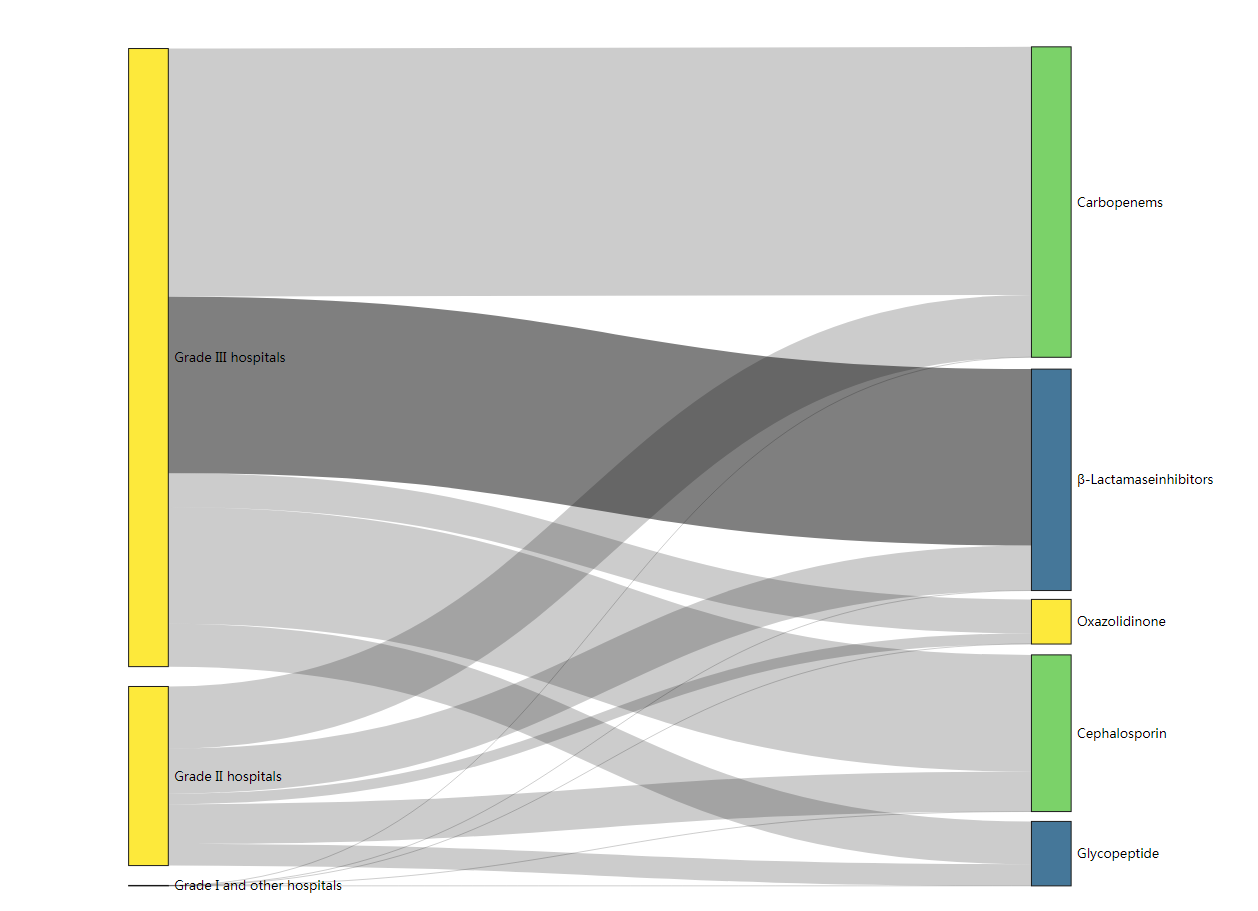

Supplement: Supplementary Figure 1 — Prescribing preferences of antibiotics among hospitals at different grades. [file Image_1.TIF]
